# Fusion of Multiple Lidars and Inertial Sensors for the Real-Time Pose Tracking of Human Motion

**DOI:** 10.3390/s20185342

**Published:** 2020-09-18

**Authors:** Ashok Kumar Patil, Adithya Balasubramanyam, Jae Yeong Ryu, Pavan Kumar B N, Bharatesh Chakravarthi, Young Ho Chai

**Affiliations:** Virtual Environments Lab, Graduate School of Advanced Imaging Science, Multimedia and Film, Chung-Ang University, Seoul 06974, Korea; ashokpatil03@cau.ac.kr (A.K.P.); adithyakoundinya@gmail.com (A.B.); puls36@cau.ac.kr (J.Y.R.); pavanbn8@gmail.com (P.K.B.N.); chakravarthi589@gmail.com (B.C.)

**Keywords:** human motion, activity recognition, position estimation, lidar, inertial sensor, motion reconstruction, locomotion, position tracking

## Abstract

Today, enhancement in sensing technology enables the use of multiple sensors to track human motion/activity precisely. Tracking human motion has various applications, such as fitness training, healthcare, rehabilitation, human-computer interaction, virtual reality, and activity recognition. Therefore, the fusion of multiple sensors creates new opportunities to develop and improve an existing system. This paper proposes a pose-tracking system by fusing multiple three-dimensional (3D) light detection and ranging (lidar) and inertial measurement unit (IMU) sensors. The initial step estimates the human skeletal parameters proportional to the target user’s height by extracting the point cloud from lidars. Next, IMUs are used to capture the orientation of each skeleton segment and estimate the respective joint positions. In the final stage, the displacement drift in the position is corrected by fusing the data from both sensors in real time. The installation setup is relatively effortless, flexible for sensor locations, and delivers results comparable to the state-of-the-art pose-tracking system. We evaluated the proposed system regarding its accuracy in the user’s height estimation, full-body joint position estimation, and reconstruction of the 3D avatar. We used a publicly available dataset for the experimental evaluation wherever possible. The results reveal that the accuracy of height and the position estimation is well within an acceptable range of ±3–5 cm. The reconstruction of the motion based on the publicly available dataset and our data is precise and realistic.

## 1. Introduction

Understanding human motion is key for intelligent systems to coexist and interact with humans. Motion tracking is a technique to track and localize the three-dimensional (3D) orientation of a human body joint [1]. Human motion tracking is widely used for activity analysis in many areas and is a current research topic due to the advancement in micro-electro-mechanical system (MEMS) sensors with wireless communication technologies [2]. Human motion tracking and recognition is a challenging problem as the human body is very flexible and has 244 kinematic degrees of freedom [3].

In recent years, scientific research has significantly emphasized pose tracking, motion capture, activity recognition, and the reconstruction of human motion. Recreating full-body human motion accurately on a 3D stick/avatar model is a challenging task. Several techniques have been proposed to capture data that can be reconstructed and recognized accurately. Pose tracking is classified into two main categories: Marker-based and Marker-less systems. Marker-based pose tracking is a traditional method where the angle between the markers placed near the joints provide the orientation and positional details of the person. The marker-based system is bounded within a geographical range of the tracking device’s field of view (FoV). Therefore, this method is only applicable in an indoor environment. Additionally, along with the prolonged setup time of the markers on the body (palpation error [4]), the markers may move due to skin stretching and suit displacement [5] contributing to errors in the reading.

The marker-less systems are emerging as more feasible and are becoming increasingly pervasive in applications that span health, arts, digital entertainment, and sports [6]. The marker-less motion capture systems (MMSs), such as depth-sensing sensors [7], are widely used for human motion tracking and reconstruction. These kinds of MMSs have disadvantages, such as limited FoV, depth, and so on, that are similar to those of marker-based systems. The depth-sensing sensors are limited to the size of the tracking volume. Due to this limitation, single-sensor approaches were mostly constrained to tracking body posture, physical therapy, rehabilitation [8], physical fitness in elderly individuals [9], ergonomics [10], anthropometry [11], and so on. Some researchers [12,13,14,15] have proposed setups with multiple depth-sensing sensors to cover a more considerable distance. For example, Müller et al. [16] used six depth-sensing camera sensors to achieve 9 m distance of tracking. However, a need exists for precise position tracking that is easy to set up, covers an extended range of distance, and is flexible to various environmental conditions.

**Figure 1:**
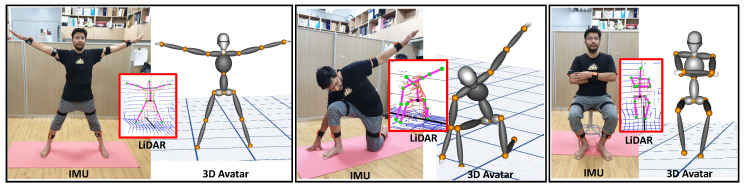
Real-time pose tracking of human motion for reconstruction on a three-dimensional model using multiple lidars and inertial measurement units (IMUs).

The implementation of the proposed system considers human body joint orientation based on the inertial measurement unit (IMU) and light detection and ranging (lidar) generated for 3D position tracking. The fusion of the sensor data is reconstructed on a virtual 3D avatar as depicted in Figure 1. The current proposed work is validated in an indoor environment.

The paper is structured as follows: Section 2 discusses the related work on various advancements in MMSs. Section 3 details the proposed system for position and orientation estimation. Next, the proposed system implementation and experimental results are presented in Section 4 and Section 5. Finally, the work is concluded with a discussion of future work.

## 2. Related Work

Several methods exist to capture and recognize human motion depending on the data capture equipment (depth cameras, IMUs, and lidars). Depth cameras are widespread primarily due to ease of use and availability of open-source tools and community [17] (e.g., the Microsoft Kinect depth camera). Depth cameras convert depth data into RGBZ data. This helps detect human joints [18] and extract rotational information from the skeletal structure. However, the methods suffer from occlusion [19]. Multiple depth sensors strategically positioned in the environment [20] can reduce the body occlusion issue but do not fully compensate for it. In [21], the accuracy of the Kinect was evaluated in terms of detecting the human body center of mass using the length of body links in the Kinect skeleton model.

Kinect is inaccurate in recognizing the center of joints while measuring short links of the body, such as the foot [22], and [23] assessed the accuracy of the Kinect in lower-extremity gait analysis. In these studies, the accuracy of the Kinect was assessed using a commercial MMS as the gold standard. They reported considerable errors in tracking ankle joint angles using both versions of Kinect, which indicates some inherent challenges in this sensor. Some recent research study [24,25,26] used machine learning-based pose estimation methods to track human pose. These method uses two-dimensional RGB cameras to recognize human motion.

The IMU sensors offer the accurate orientation of a rigid body in the form of quaternions, Euler angles, and axis angles. Quaternions are a better and gimbal-lock-free representation, unlike Euler angles [27]. Therefore, most MMSs use IMUs, capturing the data in the form of quaternions. A human body comprises various interconnected bones and joints, and it is imperative to understand and set up a hierarchical and kinematic model of a human body before attaching IMUs on a person. Thus, most of the motion databases include hierarchical information along with rotational data [28], solving the body occlusion problem.

However, IMU-based pose tracking is not mature enough to detect accurate positional data for individual joints [29] and is majorly used for motion analysis in rehabilitation and physiotherapy [30,31]. To counter this, a merger of IMU data with depth cameras has been attempted [32,33,34]. In [32], the fusion of sensors is adapted to validate the acquired movement data in two steps (generative and discriminative). In the generative process, the sensor provides human pose data, whereas the discriminative process validates the data. In other research [33], the purpose of sensor fusion is to complement each other for accurate results. In the lidar-IMU fusion experiment, the IMU sensor provides orientation information, whereas the lidar is used to filter the data. A similar approach was proposed by [34], where the IMU sensor detects human rotation and a laser sensor detects the human body position to correct the drift over time. The approach presents only the trajectory of the human motion in outdoor environment but the full skeleton pose is not described.

The current work focuses on full-body tracking with an easy multi-sensor set up (lidars and IMUs), that enables an estimation of joints’ position, bone segment orientation, and reconstruction on a 3D avatar in real-time.

## 3. Method Overview

**Figure 2:**
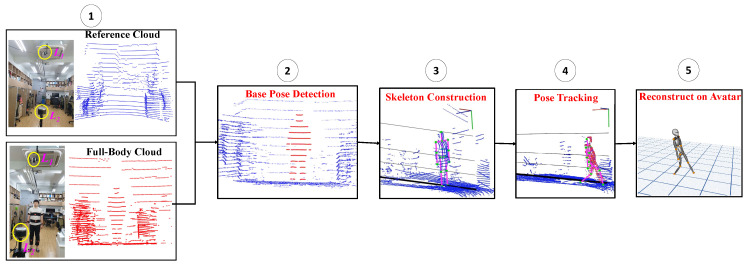
Sequence diagram of the proposed system: Acquisition → base pose detection → skeleton construction → pose tracking → reconstruction.

In this section, we discuss more details on the sensor fusion system based on lidar and IMU for position and orientation estimation and reconstruct the motion on the 3D avatar. In the proposed approach, the process of human body tracking includes the following as depicted in Figure 2: (1) Initial data retrieval (reference and full-body clouds), (2) base pose detection, (3) skeleton construction, (4) real-time pose tracking, and (5) reconstruction using the avatar.

**Figure 3:**
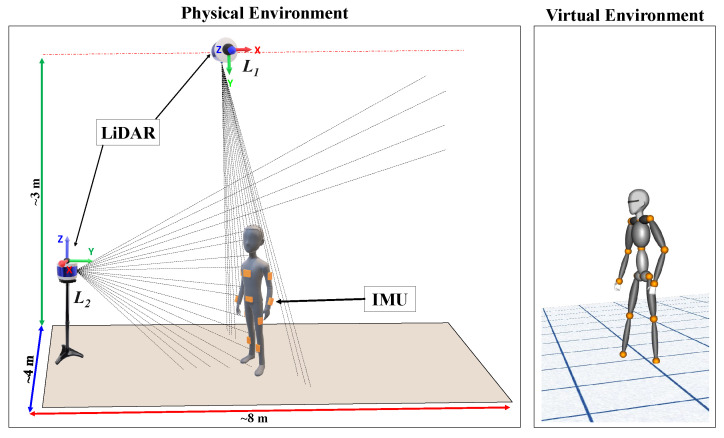
Proposed light detection and ranging (lidar)-IMU fusion-based system for human motion tracking in an indoor environment.

In the proposed method, two 3D lidar sensors were used to track the position of the human body, and IMU sensors were used to estimate the orientation and position of each joint during the human body activity in real time. Figure 3 illustrates the complete setup of multiple lidars and IMUs (the laser ray depicted in the figure is only in *y* direction of lidar, i.e., the vertical FoV).

The 3D lidar-based human body tracking process includes the segmentation of raw data and the classification of objects of interest. The lidar sensor used in this system has a distance range and accuracy of up to 100 m and ±3 cm. For human body tracking, the maximum range is 14 to 17 m [35], which is well within the 100 m range. Therefore, in the current work, two lidars ($L_{1}$ and $L_{2}$) were used within an operating range of an 8 × 4 × 3 m indoor environment (Figure 3).

### 3.1. Initial Data and Pose Extraction

To track the user in real time, two separate sets of point cloud data ($P_{\left(i\right)}\left\{x,y,z\right\}$, where i=0 to *n* point data) were initially acquired in the calibration step. One set, with the user ($Pf_{\left(i\right)}\left\{x,y,z\right\}$; full-body cloud) in the FoV, primarily computes the actual height of the user and constructs the skeleton structure. The second set of point cloud data, without the user ($Pr_{\left(i\right)}\left\{x,y,z\right\}$; reference cloud) in the FoV, filters the user from the background data. The reference cloud has information about the environment in which human motion is detected. To compute the actual height and construct a skeleton structure, the user must stand at an optimal distance away so that both lidars covers the full body (Figure 3) within their collective FoVs.

Thus, the acquired full-body cloud was compared against the reference cloud to extract the position of the user point cloud in a real environment (*x*, *y*, and *z*-axes). An Octree-based change detection algorithm [36] was adopted to filter out the user point cloud ($P_{t(i)}\left\{x,y,z\right\}$) from the full-body cloud, as depicted in the second step of Figure 2. The main aim in this section is to extract the point cloud corresponding to the user and the accuracy of the extraction directly affects the following process and correctness of the result.

The ground point g{x,y,z} is the actual floor location from $L_{1}$, considering the maximum point in the *y*-axis, as illustrated in Figure 3. A slight inclination occurs in $L_{1}$ due to its mounting. Therefore, the resulting point cloud has an inherent slope (*m*). Considering the actual floor is at *g*, the slope *m* is given by Equation (1).

(1) $$m=\left(max_{y}-g_{y}\right)/\left(max_{x}-g_{x}\right),$$

where $g_{x}$ and $g_{y}$ are the *x* and *y* component of *g*, and $max_{x}$ and $max_{y}$ are the maximum *x* and *y* component of $P_{t}$.

The user may be located at any point on this slanted floor. Therefore, the slope of the floor due to the inclination of $L_{1}$ should be factored into computing the actual height ($A_{h}$) of the user, as indicated in Equation (2):

(2) $$A_{h}=\left(g_{y}-min_{y}\right)+c_{x}\times m,$$

where $min_{y}$ is the minimum *y* component of $P_{t}$, and $c_{x}$ is the *x* component of the centroid of $P_{t}$.

### 3.2. Identifying the Human Skeletal Structure

**Figure 4:**
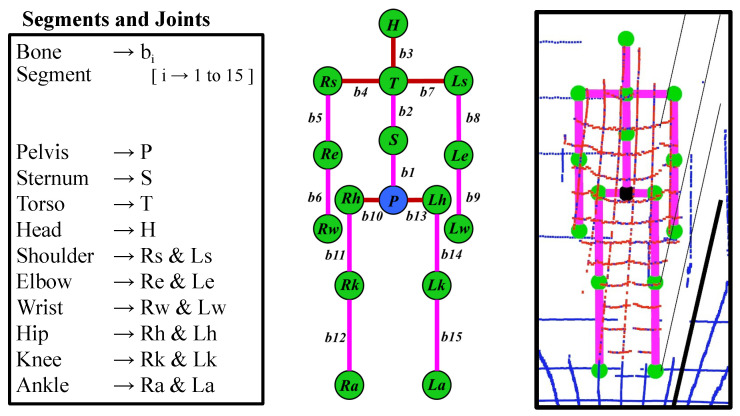
Generated human skeleton using the three-dimensional lidar point cloud.

The maximum ($max_{y}$) and minimum ($min_{y}$) in the $P_{t}$ provide the actual height of the user with an accuracy of ±3–5 cm. The actual height (see Equation (2)) is the baseline for calculating each body part proportion to construct the skeleton of the user. An average person is generally 7.5 times the height of his or her head [37]. To construct each bone segment in the skeleton, we considered the head height ($H_{h}$) to be the standard measurement proportion (i.e., $H_{h}=A_{h}/7.5$), which is used to parameterize the lengths of each segment [38]. Figure 4 depicts the constructed skeleton from the point cloud with 15 segments ($b_{1}$ to $b_{15}$) and 16 connecting joints. At this step, we know the relative joint positions of the human skeleton, which aids in the estimation of real-time pose tracking.

### 3.3. Real-Time Pose Tracking

We captured the initial position and generated the human skeleton in the previous subsections. Along the same lines, the movement of the user in real time was acquired (position) as the person moves from the initial position. In the current work, the real-time motion of the full-body position and orientation was estimated using 10 IMU sensors attached to the human body (bone segments, Figure 3). Concurrently, the pose from the lidar data was estimated and fused with the IMU sensor data because the pose estimate of the IMU sensor is affected by the displacement drift. In the following section, we discuss more details regarding the position and orientation estimation.

#### 3.3.1. Position and Orientation from Inertial Sensors

**Figure 5:**
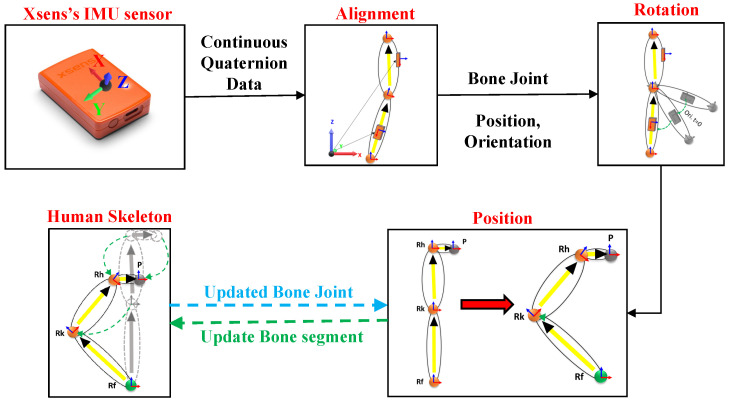
Process of updating the bone joint position and orientation.

The IMU sensors were used to estimate body segment position and orientation changes in real time (segments are connected by joints), and the changes were updated on a biomechanical model of avatar segments. The IMU sensors used in our work output orientation data in the form of quaternions ($q=(q_{w},q_{x},q_{y},q_{z})$). Full-body motion was captured over time for 10 joint-bone segments. Moreover, the orientation of 5 segments (red color in Figure 4) rely on the torso (i.e., $b_{3}$, $b_{4}$, and $b_{7}$) and pelvis (i.e., $b_{10}$ and $b_{13}$) bone joint sensors. All segments are hierarchically connected in the avatar, as presented in Figure 4. The position and orientation of the joint estimation process are illustrated in Figure 5.

**Figure 6:**
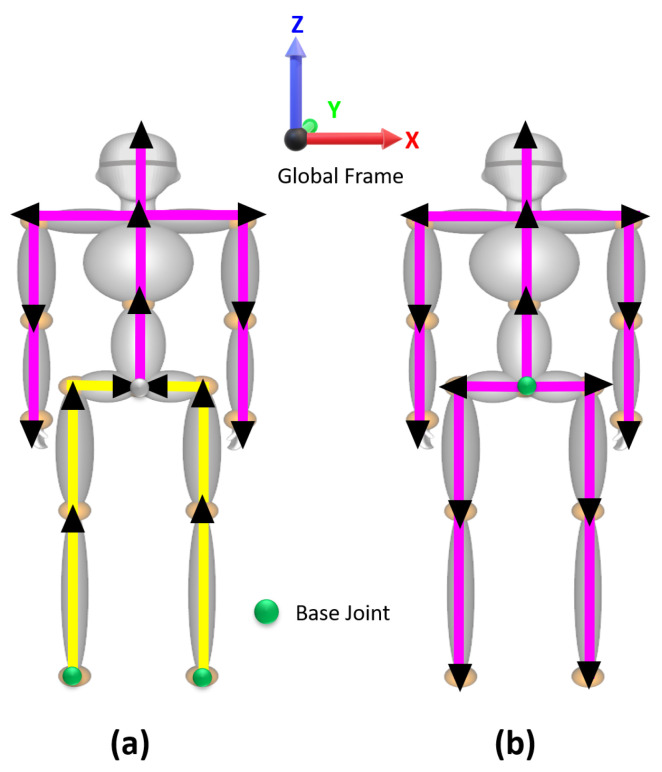
Estimating position using lower limb rotation from inertial measurement unit (IMU) sensors: (**a**) Updating from foot position to pelvis and (**b**) updating from pelvis to full body.

The IMU sensors provide the orientation with respect to a global coordinate frame (*x*-axis pointing north, *z*-axis against gravity, and *y*-axis pointing west). For each bone segment, all kinematic parameters were expressed in a common coordinate global frame, which is the right-handed Cartesian coordinate system (Figure 6). The sensors were calibrated and aligned to the global frame to compute the rotation of the individual joint-bone segment, as given in Equation (3):

(3) $$Aq_{i}=q_{i}q_{0}^{-1};\left[i=0\;to\;n\;frames\right],$$

where $q_{i}$ is the continuous frames of quaternion data from the IMU sensors, $q_{0}^{-1}$ is the inverse of first $q_{i}$, and $Aq_{i}$ denotes the aligned quaternion data.

After the alignment of the sensors to the global frame, the joint position and segment rotation were computed. We considered each joint position to be a unit vector in the direction parallel to the respective bone axis in the attention pose (Figure 6). For instance, if we consider the foot joint axis parallel to the *z*-axis, then a unit vector for the foot joint can be determined as $\hat{v}=\left(0,0,1\right)$, and it is represented as $q_{v}=\left(0,0,0,1\right)$ in quaternion form:

(4) $$Rv=Aq_{i}\times q_{v}\times Aq_{i}^{-1},$$

where Rv is the rotated joint vector after quaternion multiplication in quaternion form.

Next, we extracted the rotated vector from $R_{v}=\left(q_{w},q_{x},q_{y},q_{z}\right)$ (i.e., joint vector $\hat{J_{v}}=\left(q_{x},q_{y},q_{z}\right)$) and updated the respective joint position in the skeleton by considering the neighbor joint, scaling it to the respective segment length as given in Equation (5):

(5) $$P_{cJoint}=P_{nJoint}+\hat{J_{v}}\times S_{length},$$

where $P_{cJoint}$ is the updated position of the current joint, $p_{nJoint}$ is the position of the neighboring joint to $P_{cJoint}$, and $S_{length}$ is the length of the respective bone segment.

Figure 6 illustrates an overview of the positional relation of the bone joints with the adjacent joint, denoted by a directional vector (describing the unit vector at that joint with its direction) with the length of the individual segments. In Figure 6a,b, the joints marked in green are the base joints, as the positions of the rest of the joints in the lower body are dependent on these joints (right foot ($R_{f}$) and left foot ($L_{f}$)). The positions of all upper body joints are dependent on the pelvis position (*P*). Magenta and yellow indicate the individual bone segments. The magenta indicates the directional vector starts from the pelvis as the base position, whereas yellow presents the directional vector begin from the foot as the base position.

**Figure 7:**
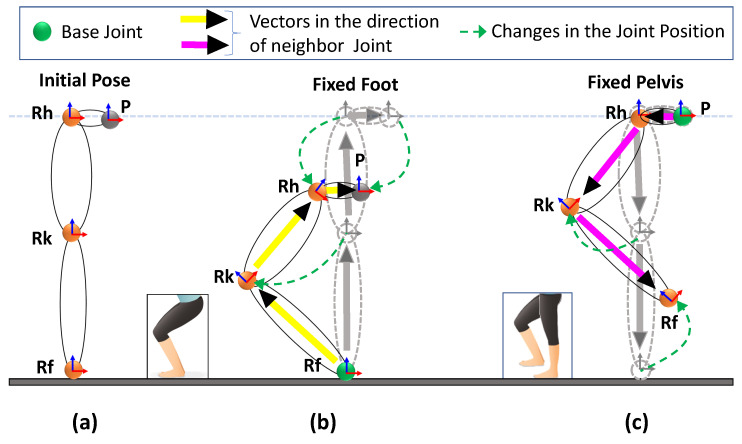
Computing the pelvis position. (**a**) Position of lower right leg joints in the attention pose, (**b**) updating knee, hip, and pelvis positions with the fixed foot position (bottom-up update), and (**c**) updating the hip, knee, and foot position with the fixed pelvis (updated from (**b**)) position (top-down update).

For instance, in Figure 7b, the upper body is moving vertically down due to the rotation in the leg. In this condition, the positions of the other joints were estimated by considering the fixed foot joint ($R_{f}$) to be a reference point (base position), and all joint positions were updated (bottom-up joint position update). Similarly, in Figure 7c, the pelvis joint is fixed, and the foot is changing. The pelvis is the reference point (base position), and all joint positions were updated (top-down joint position update).

#### 3.3.2. Position Tracking from Lidars

With the efficient extraction of the base position in the initial stage (Section 3.1), locating the real time position using lidar data has two simple steps. The first step is extracting the full-body cloud ($P_{tr}$) of a user in real time (similar to the procedure followed in Section 3.1). The second step is detecting all bone segments by their geometry using the particle filter [39] and tracking only the legs to locate the real-time foot positions. The detected foot positions are aided to correct the displacement drift in the positions calculated using the bone orientation.

In the $P_{t}$ data (Section 3.1), the point clouds corresponding to the lower leg ($P_{t}^{leg}$) are clustered with the aid of the joint positions and bone segment lengths. Our approach employs a similar technique to detect the leg position in the point clouds, as proposed by [34]. In the current work, we used a particle filter [39] to track the lower leg-bone point cloud. The particle filter tracks the observation cloud ($P_{t}^{leg}$) within the measured point cloud ($P_{tr}$). Thus, the computed foot positions from the output of the particle filter are used to correct the displacement drift within a threshold distance (δ=10 cm).

## 4. Implementation Details

Our proposed system consists of the following sensory setup and calibration steps.

(1) Velodyne VLP-16 lidar: A Velodyne lidar is used to estimate the initial position and height of the subject and to track the real-time position. It offers 16-channel lidar scanning with a $360^{\circ }$ horizontal and $\pm 15^{\circ }$ vertical FoV, as illustrated in Figure 3. The sensor has low power consumption, scans the environment in three dimensions at a frequency of 10 to 20 Hz, and generates 600,000 points per second with a maximum range of 100 m with a claimed accuracy of ±3 cm. Due to the frequency difference between lidars and IMUs, we adopted a linear interpolation of the positional data of the lidar to match the IMU body orientation data.

To obtain a dense point cloud, two lidars are perpendicularly positioned, as presented in Figure 3. The lidar at the top ($L_{1}$) is used to track a person from the top view (which primarily aids in tracking the position when a person poses parallel to the ground (sleeping condition)), and is also used to estimate the height of a person (with an error within ±3 to 5 cm) and the ground position (floor). Another lidar is used to create dense point data that are located on the front side of the user. To integrate multiple lidar data into a single frame, we followed the procedure in the work by [40]. The normal distributions transform (NDT) algorithm [39] is used for point cloud registration.

(2) Xsens IMU: The MTw motion tracking system is a miniature IMU [41] (Figure 5). It is a small, lightweight, wireless inertial sensor-based 3D motion tracker manufactured using MEMS technology. This sensor returns the 3D orientation, acceleration, angular velocity, static pressure, and earth-magnetic field intensity at a frequency of 60 Hz. Only the 3D orientation is considered for the proposed work. The real-time motion of the full-body position and orientation is estimated using 10 IMU sensors attached to the human body segments, except $b_{3}$, $b_{4}$, $b_{7}$, $b_{10}$, and $b_{13}$, as shown in the skeleton structure with the maroon color. Before tracking and capturing the data, the sensors must be calibrated to avoid the incorrect estimation of the base position and to reduce sensor drift. These issues lead to the misalignment of the bone segments, which results in the mismatching of the avatar to the user in real time. The calibration routine has one step with an attention pose.

## 5. Experiments

We conducted an experimental evaluation of the proposed fusion system by considering various poses (involving changes in full-body joint position and segment orientations) by conducting a statistical analysis of the acquired real-time data. Multiple key poses were considered, which affect multiple joint segments, both in position and orientation. The first objective is to investigate the accuracy of the proposed fusion system concerning the position estimation against the ground truth. The second objective is to compare the proposed system against a publicly available 3D pose estimation dataset, the TotalCapture dataset [42].

### 5.1. Height Accuracy

The accuracy of the joint position highly depends on the length of the bone segments, as a result of the user’s height computed from the lidar data, as described in Section 3.1. The heights (estimated) of seven different users are compared against their known actual heights (ground truth). Considering the inherent error in the lidar and the error due to mounting, an error of ±3 cm in the calculated height is shown in Table 1. As $H_{h}$ is the standard measurement proportion for skeleton construction, the error in the length of individual segments trickles down to less than 1 cm. Therefore, this difference in the height is insignificant for the construction of the skeleton and has a minimal effect on the position estimation of joints.

### 5.2. Orientation Accuracy

**Figure 8:**
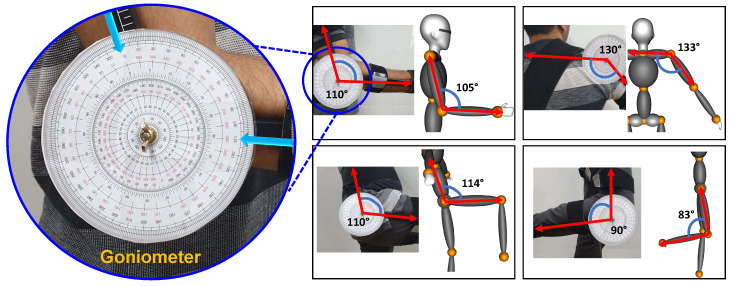
Comparison of reconstruction angle between two bone segments against ground truth.

To provide the validation for orientation accuracy of motion reconstruction on the avatar, we compare against the ground truth angle data. To formulate ground truth angle data, we selected a physically measurable angle between two bone segments using measurement apparatus (Goniometer) [43] as highlighted and depicted in Figure 8. Few common poses are chosen for different bone segments and manually noted as ground truth angle data. Simultaneously IMU sensors are attached and orientation data (quaternion) is recorded from respective bone segments. The angle between two bone segments is estimated (i.e., inverse cosine of the dot product of two quaternions) and compared against the ground truth values as shown in Figure 8. The estimated mean error of the measured angle is within the ±5° for the proposed system.

### 5.3. Full-Body Position Accuracy

**Figure 9:**
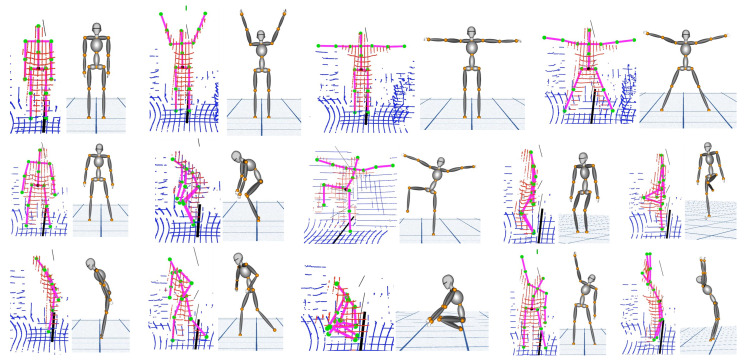
Overlapping point cloud and stick model indicate the accuracy of orientation and position. The same is reconstructed on the three-dimensional avatar for 14 different key poses.

**Figure 10:**
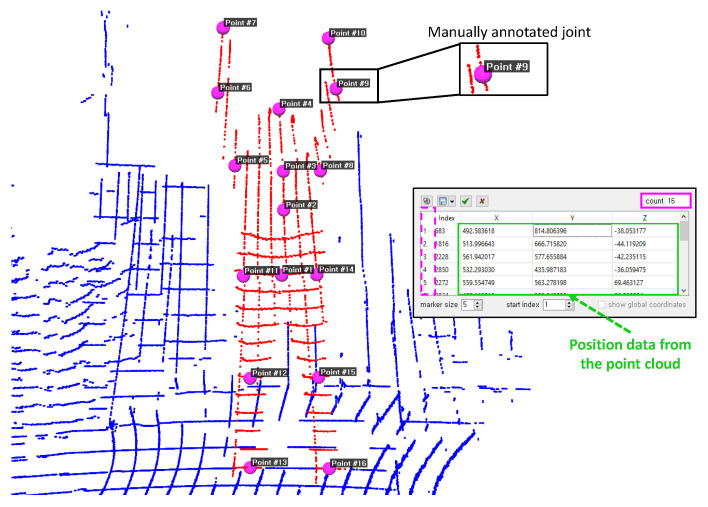
An example demonstrating manually annotated position data using the CloudCompare tool for validating position accuracy.

To validate position accuracy, 14 different poses that affect all 16 joint positions were considered, as indicated in Figure 9. The point cloud data captured from the lidar have positions corresponding to different joints. The data are manually annotated for 14 different poses using the CloudCompare tool (3D point picking list feature) [44], as depicted in Figure 10. The labeled data are used as the ground truth for measuring the accuracy of the estimated joint position. Figure 9 presents a visual comparison of the reconstruction of poses against the ground truth, which is a reasonably realistic reconstruction.

**Figure 11:**
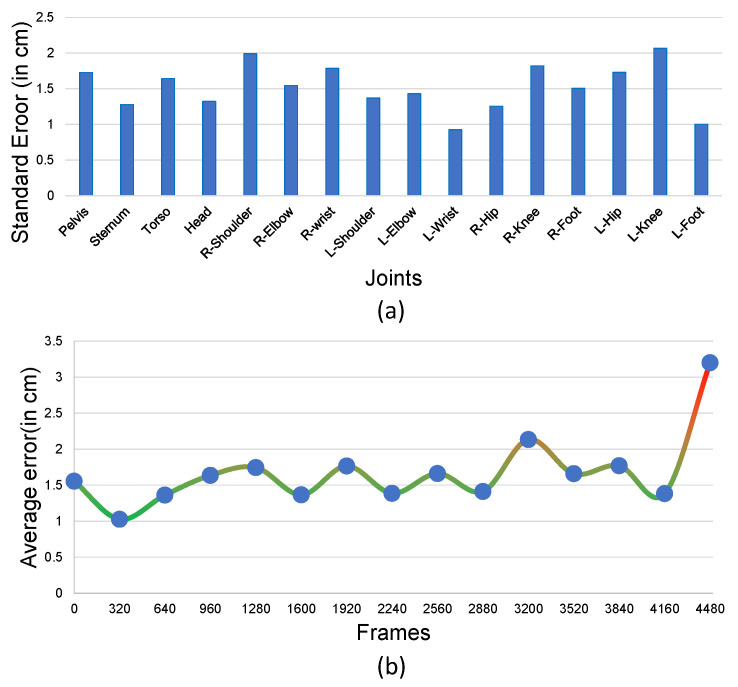
(**a**) Standard error in the position with respect to the ground truth (in cm) for 14 poses and (**b**) average positional error for all joints for 14 poses for over 4480 frames (60 fps).

The Figure 11 demonstrates the 14 different poses captured at 60 fps, with a total of 4480 frames. The standard error in the position for individual joints and the error in the position concerning the ground truth were both well within 5 cm, as depicted in Figure 11a. Figure 11b displays the linear average positional error for all joints over time.

**Figure 12:**
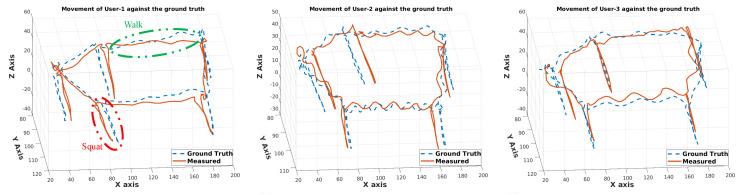
Proposed fusion-based trajectory against the ground truth (in cm) for the walk and squat pattern.

The calculated pelvis position was compared to the ground truth. Three different users with different heights performed a combination of walking and squatting movements in a predefined geometric pattern. The changes in the pelvis position for all users in the three axes are revealed in Figure 12. The pelvis position in a geometric pattern with intermediate squats in five different locations was observed with an accuracy of up to ±5 cm in all conditions.

### 5.4. Position Estimation Using the Total Capture Dataset

The TotalCapture dataset [42] contains orientation information acquired from multiple Xsens IMU sensors attached to bone segments. Joint position data were acquired from a multiple viewpoint video. Various motions, such as walking, acting, and freestyle, and range of motion (ROM) were available as part of the dataset. For the current study, we considered multiple movements within the orientation data that affect all joints. The positions of joints were estimated using the proposed method and were compared against the position data in the TotalCapture dataset. Table 2 lists six different motion types, the respective observed joints, the standard deviation, and the mean difference from the ground truth. The results reveal that the estimated positions are at an average standard deviation of 0.24 cm and an average mean difference of 0.86.

### 5.5. Accuracy of Reconstruction on the Avatar

**Figure 13:**
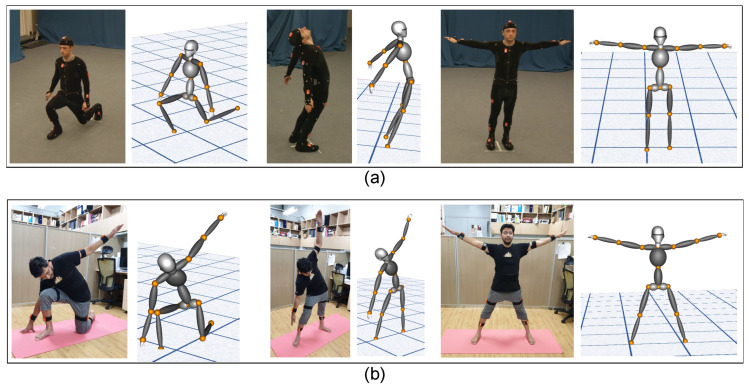
Validation of motion reconstruction on the three-dimensional avatar: (**a**) Reconstruction of the TotalCapture range of motion (Subject 1) data and (**b**) reconstruction of the user data.

The system estimates bone segment orientation in 3D and full-body joint positions using IMU and lidar sensor data fusion. This enables users to track their pose while performing motion in real time. In this section, we validate the accuracy of our 3D model for motion reconstruction. The 3D model avatar was developed using a visualization toolkit (in C++) [45]. Section 3.3.1 details how the model is updated. The TotalCapture dataset has various ROM, which were applied directly to the 3D avatar to validate the reconstruction accuracy. Figure 13a presents a few selected reconstructed poses from the TotalCapture dataset against their ground truth images. Figure 13b shows multiple poses reconstructed on the same model using our data against the ground truth images. The results reveal that the reconstruction is reasonably accurate.

## 6. Discussion

In the previous section, the results demonstrate that the pose tracking of human motion with the estimation of the orientation and position is reasonably accurate and within the range of ±3–5 cm. The position estimation of the pelvis using the lower body orientation and the estimation of the full-body joint position is an effective approach. The reconstruction of the motion on the 3D avatar is realistic and delivers results comparable to state-of-the-art pose-tracking systems, such as TotalCapture [42]. The position of the foot is continuously corrected for displacement drift due to the position estimation from the lower body orientation. Furthermore, the approach uses fewer sensors with a relatively easier installation setup and has minimal environmental dependencies. We use a simple calibration where the user starts at an attention position. The proposed system can be adopted for real-time pose-tracking applications, such as in rehabilitation, athletic performance analysis, surveillance, human-machine interfaces, human activity tracking and recognition, kinesiology, physical fitness training and therapy, human-computer interaction, virtual reality, and so on.

Nevertheless, during the bottom-up update, while estimating the pelvis position from the fixed foot, the right and left legs were translated to the ground before computing the pelvis position. As the foot positions are fixed at every step on the ground and the right and left legs are independently considered, human activities involving jumping, running, and locomotion, such as hand walking, cannot be reconstructed realistically on the 3D avatar. During such activities, the avatar suffers occlusion with the ground. To counter such issues, multiple kinematics and rigid body constraints can be applied to the model, and acceleration from the IMU sensors could be used to estimate the position of the joints to increase the efficiency and accuracy of the system.

## 7. Conclusions

The results of our experimental evaluation demonstrated that the overall lidar and IMU fusion-based system exhibited better accuracy in estimating the joint position and bone segment orientation. The experimental setup of the proposed system was relatively more accessible and flexible concerning sensor locations.

The proposed method was efficient and accurate for human pose-tracking system by fusing lidar and IMU sensors. The system estimated body joint orientation and position in 3D using IMU sensors and used lidars to compensate for the displacement drift. The lidar data were also instrumental during the initial calibration and user height estimation for skeleton construction.

The TotalCapture dataset is used wherever possible for validating the proposed approach and the accuracy of the reconstruction on the 3D model. Multiple experiments were conducted to validate the proposed system against the ground truth. All results indicated that the proposed system could be used in real-time applications as stated above. Future work involves the consideration of complex human activities, such as running, jumping, hand walking, dancing, and so on that have more spatio-temporal changes in the orientation and position of the bone segments and joints. 

## Figures and Tables

**Table 1:** Accuracy of user height estimated from the lidar data against the ground truth (in cm).

Person	Ground Truth Height	Lidars Calculated Height	Change in Height	Error
1	180	178	−2	
2	168	165	−3	
3	171	172	1	
4	155	158	3	SD = 0.44,
5	175	178	3	Mean = 2.7
6	164	162	−2	
7	161	164	3	

**Table 2:** Positional difference (in cm) between TotalCapture [42] and our proposed method for a few selected motions in the TotalCapture dataset. (* See Figure 4 for abbreviations).

Sl. No.	Motion Type	Frame No.	Observed Joints	Standard Deviation	Mean Difference
1	Upper Arm Swing	292–351	Rs, Re, Rw, Ls, Le, Lw	0.02	0.45
2	Upper Arm Rotation	745–818	Rs, Re, Rw, Ls, Le, Lw	0.08	0.36
3	Lower Arm Swing	1235–1300	Re, Rw, Le, Lw	0.02	0.33
4	Pelvis Bending	2475–2389	P, S, T, H	0.21	0.73
5	Right Upper Leg Swing	3200–3250	Rh, Rk, Ra	0.40	0.98
6	Squat	4703–4836	Full Body	0.68	2.35

## Abbreviations

The following abbreviations are used in this manuscript:

lidar	Light Detection and Ranging
IMU	Inertial Measurement Unit
FoV	Field of View
MMS	Marker-less Motion Capture System
ROM	Range of Motion
